# Supplementary material for: Prevalence and factors associated with comorbidities in Iranian patients with type 2 diabetes: A national study
Source: PLoS One. 2026 Feb 27;21(2):e0343690. doi: 10.1371/journal.pone.0343690 (PMC12948068; doi:10.1371/journal.pone.0343690)
Supplement: S1 Fig — (DOCX) [file pone.0343690.s001.docx]

**S1 Fig. The proportion of missing values for each variable utilized in the study**
