# Supplementary material for: Prevalence and factors associated with comorbidities in Iranian patients with type 2 diabetes: A national study
Source: PLoS One. 2026 Feb 27;21(2):e0343690. doi: 10.1371/journal.pone.0343690 (PMC12948068; doi:10.1371/journal.pone.0343690)
Supplement: S1 Table — (DOCX) [file pone.0343690.s002.docx]

**S1 Table.** Stepwise Multinomial Logistic Regression Analysis of Factors Associated with Comorbidity in the Female Diabetic Population

| variables | **1 comorbidity vs. 0 comorbidity** | | **2 comorbidities vs. 0 comorbidity** | |
| --- | --- | --- | --- | --- |
|  | **OR (95% CI)** | **P value** | **OR (95% CI)** | **P value** |
| Age (years) |  |  |  |  |
| - < 60 | Reference | | Reference | |
| - ≥ 60 | **2.03 (1.36, 3.04)** | **0.001** | **5.55 (3.58, 8.60)** | **< 0.001** |
| Physical activity (min/week) |  |  |  |  |
| - < 150 | Reference | | Reference | |
| - ≥ 150 | **0.68 (0.48, 0.96)** | **0.029** | **0.54 (0.36, 0.83)** | **0.004** |
| Years of schooling (years) |  |  |  |  |
| - <6 | Reference | | Reference | |
| - 1_6 | 0.66 (0.45, 1.01) | 0.053 | **0.52 (0.32, 0.85)** | **0.009** |
| - 7_12 | **0.58 (0.34, 0.99)** | **0.046** | 0.51 (0.23, 1.12) | 0.096 |
| - >12 | **0.52 (0.31, 0.89)** | **0.017** | **0.29 (0.17, 0.52)** | **< 0.001** |
| BMI (kg/m²) |  |  |  |  |
| - <25 | Reference | | Reference | |
| - 25-30 | 1.46 (0.84, 2.52) | 0.179 | 1.15 (0.57, 2.31) | 0.703 |
| - >30 | **1.80 (1.05, 3.08)** | **0.031** | 1.95 (0.96, 3.93) | 0.063 |
| OR, odds ratio; CI, confidence interval; BMI, body mass index.  Data have been weighted to account for overall non-response, non-response at each step, and sample distribution across provinces, adjusted for age, sex, and area of residence.  Stepwise selection was used for variable selection, with an entry p-value of 0.2 and a removal p-value of 0.05.  Statistically significant (P value<0.05) statistics are bold. | | | | |
