# Supplementary material for: Prevalence and factors associated with comorbidities in Iranian patients with type 2 diabetes: A national study
Source: PLoS One. 2026 Feb 27;21(2):e0343690. doi: 10.1371/journal.pone.0343690 (PMC12948068; doi:10.1371/journal.pone.0343690)
Supplement: S2 Table — (DOCX) [file pone.0343690.s003.docx]

**S2 Table.** Stepwise Multinomial Logistic Regression Analysis of Factors Associated with Comorbidity in the Male Diabetic Population

| variables | **1 comorbidity vs. 0 comorbidity** | | **2 comorbidities vs. 0 comorbidity** | |
| --- | --- | --- | --- | --- |
|  | **OR (95% CI)** | **P value** | **OR (95% CI)** | **P value** |
| Age (years) |  |  |  |  |
| - < 60 | Reference | | Reference | |
| - ≥ 60 | **1.99 (1.11, 3.60)** | **0.022** | **4.43 (2.30, 8.54)** | **< 0.001** |
| Physical activity (min/week) |  |  |  |  |
| - < 150 | Reference | | Reference | |
| - ≥ 150 | **1.54 (1.01, 2.34)** | **0.044** | 1.03 (0.64, 1.64) | 0.911 |
| Residential area |  |  |  |  |
| - Urban | Reference | | Reference | |
| - Rural | **1.97 (1.28, 3.02)** | **0.002** | 1.48 (0.90, 2.43) | 0.118 |
| Years of schooling (years) |  |  |  |  |
| - <6 | Reference | | Reference | |
| - 1_6 | 0.59 (0.31, 1.12) | 0.105 | 0.63 (0.34, 1.18) | 0.151 |
| - 7_12 | **0.31 (0.14, 0.65)** | **0.002** | 0.47 (0.21, 1.06) | 0.069 |
| - >12 | **0.30 (0.15, 0.62)** | **0.001** | **0.44 (0.21, 0.92)** | **0.029** |
| BMI (kg/m²) |  |  |  |  |
| - <25 | Reference | | Reference | |
| - 25-30 | 1.24 (0.76, 2.03) | 0.391 | 1.39 (0.77, 2.53) | 0.279 |
| - >30 | **2.56 (1.30, 3.91)** | **0.004** | **2.28 (1.18, 4.40)** | **0.014** |
| Employment status |  |  |  |  |
| - Unemployed & retired & unpaid job | Reference | | Reference | |
| - Employed | **0.53 (0.31, 0.90)** | **0.020** | **0.33 (0.18, 0.60)** | **< 0.001** |
| OR, odds ratio; CI, confidence interval; BMI, body mass index.  Data have been weighted to account for overall non-response, non-response at each step, and sample distribution across provinces, adjusted for age, sex, and area of residence.  Stepwise selection was used for variable selection, with an entry p-value of 0.2 and a removal p-value of 0.05.  Statistically significant (P value<0.05) statistics are bold. | | | | |
