# Supplementary material for: Prevalence and factors associated with comorbidities in Iranian patients with type 2 diabetes: A national study
Source: PLoS One. 2026 Feb 27;21(2):e0343690. doi: 10.1371/journal.pone.0343690 (PMC12948068; doi:10.1371/journal.pone.0343690)
Supplement: Attachment — Submitted filename: Response to Editor.pdf [file pone.0343690.s006.pdf]

Response to Editor

Khem Descatamiento

Thank you for your consideration.

Please also provide a non-author point of contact (e.g., data access committee, ethics committee, or other institutional body) where data requests may be made. Note that it is not acceptable for the authors to be the sole named individuals responsible for ensuring data access.

Response: I Changed data availability in manuscript and provide a non-author point of contact.

The data supporting this study are not publicly available due to ethical restrictions and the presence of potentially identifiable patient information, as mandated by the Ethics Committee of the Endocrinology and Metabolism Research Institute (EMRI), Tehran University of Medical Sciences. Data may, however, be made available upon reasonable request to the Data Access Committee at the National Institute for Health Research (NIHR), Tehran University of Medical Sciences (nihr@tums.ac.ir). Requests must include a detailed research proposal and will be evaluated by the NIHR Data Access Committee in line with institutional and ethical guidelines; approved access may require a formal Data Use Agreement. The NIHR Data Access Committee—not the authors—is the designated
