# Supplementary material for: Prevalence and factors associated with comorbidities in Iranian patients with type 2 diabetes: A national study
Source: PLoS One. 2026 Feb 27;21(2):e0343690. doi: 10.1371/journal.pone.0343690 (PMC12948068; doi:10.1371/journal.pone.0343690)
Supplement: Attachment — Submitted filename: Response.docx [file pone.0343690.s005.docx]

**Thank you so much for your attention. The manuscript was checked and changes were performed. In addition, the responses to the comments are included in this file.**

**Kind Regards
Dr. Nazila Rezaei
Corresponding author**

---------------------------------------------------------------------------------------------------------------

**PONE-D-25-25729 - [EMID:3b8aced2f935fb44]**

**Prevalence and Factors Associated with Comorbidities in Iranian Patients with Diabetes: Insights from the 2021 STEPS Survey**

**Editor's comments:
Dear Dr. Rezaei,**

**We've checked your submission and before we can proceed, we need you to address the following issues:**

**1. Please report in the Methods section the dates when data were accessed for research purposes. Please note that if this information is not included when your manuscript is resubmitted, it may be rejected.**

**Response: Thank you for your comment. The sentence regarding the timing of data access has been added in line 169 on page 8. The added sentence has been highlighted.**
