# Supplementary material for: Prevalence and factors associated with comorbidities in Iranian patients with type 2 diabetes: A national study
Source: PLoS One. 2026 Feb 27;21(2):e0343690. doi: 10.1371/journal.pone.0343690 (PMC12948068; doi:10.1371/journal.pone.0343690)
Supplement: Attachment — Submitted filename: Review .docx [file pone.0343690.s004.docx]

**Review**

I reviewed the manuscript entitled **“Prevalence and Factors Associated with Comorbidities in Iranian Patients with Diabetes: Insights from the 2021 STEPS Survey”.** It is an interesting topic. The manuscript is in the scope of the journal, but certain shortcomings should be addressed before the article can be published:

- The manuscript should be edited for language, as it contains several grammatical mistakes.
- In the abstract, Add a brief info on inclusion and exclusion.
- Several abbreviations such as line 80 (T2D) and STEPS each abbreviation should be explained first then be used in the article.
- The results section needs more explanation.
- As it is requesting to publish in an international multidisciplinary Journal, in the conclusion you should include how the findings of this study can affect the general practice except for Iran ethnic groups population and some insight on future studies. The generalizability of the results should also be discussed.
