# Supplementary material for: Prevalence and factors associated with comorbidities in Iranian patients with type 2 diabetes: A national study
Source: PLoS One. 2026 Feb 27;21(2):e0343690. doi: 10.1371/journal.pone.0343690 (PMC12948068; doi:10.1371/journal.pone.0343690)
Supplement: Attachment — Submitted filename: Response to Reviewers.docx [file pone.0343690.s008.docx]

**We would like to sincerely thank the reviewers for their careful reading of our manuscript and for their insightful and constructive comments. We have revised the manuscript accordingly, and our point-by-point responses are provided below.**

**Kind Regards**

**Dr. Nazila Rezaei,
Corresponding author**

**Title:** Prevalence and Factors Associated with Comorbidities in Iranian Patients with Diabetes: Insights from the 2021 STEPS Survey

**Submission ID:** PONE-D-25-25729R1

Reviewer #3: This study analyzes nationally representative data from the 2021 Iran STEPS survey to assess the prevalence and determinants of comorbidities among adults with diabetes. The topic is timely and of clear public-health relevance, particularly given the growing burden of diabetes and associated conditions in low- and middle-income settings. The manuscript offers useful descriptive evidence and fills an important regional data gap; however, several methodological and interpretive issues.

Comments:
1. Methods: Citation [11] appears to refer to a study that utilized the Iran STEPS survey rather than a primary description of the survey methodology itself. It would strengthen the manuscript if the authors could include an appendix briefly outlining how the STEPS survey was designed and implemented. For example, the U.S. MEPS data provide a dedicated summary of the Panel Design and Data Collection Process (<https://meps.ahrq.gov/mepsweb/survey_comp/hc_data_collection.jsp>), which helps readers understand how the survey was conducted. The authors are also encouraged to report the overall response rate of the 2021 Iran STEPS survey, in addition to the number of individuals ultimately included in the analysis. The authors mention survey weights were “adjusted for non-response within age groups” and “poststratification weighting by age, sex, and residence”, but they do not specify the base weights, how they were derived from the cluster sampling, or how province-level sampling probabilities were handled. It’s unclear if they accounted for stratification and clustering in variance estimation. These should be provided in appendix.

**Response:** We appreciate the reviewer’s careful attention to the methodological details. We have revised the manuscript to incorporate the requested clarifications and enhance transparency. We would like to clarify that Reference 11 in our manuscript (in the revised manuscript reference 10) is the official study protocol of the 2021 Iran STEPS survey, which provides the primary description of the survey design. This protocol includes detailed information on the sampling strategy, target population, fieldwork procedures, and data-collection processes (DOI: *10.34172/aim.2022.99*). Furthermore, the supplementary material accompanying the protocol provides a comprehensive explanation of the weighting methodology, including computation of base weights, non-response adjustments, and post-stratification procedures. The supplementary file is publicly available at:
<https://pmc.ncbi.nlm.nih.gov/articles/instance/10685773/bin/aim-25-634-s002.pdf>. As outlined in this supplementary document, the weighting strategy explicitly accounts for the design effect, and variance estimation incorporates clustering, consistent with the complex multistage sampling framework of STEPS 2021. Because these methodological details have already been thoroughly documented in the official protocol and its supplementary material, we did not repeat them in our manuscript or its appendix. However, to enhance clarity and address your specific points, we have made the following additions to the manuscript:

1. In the Methods section (Page 6, Lines 115-116), we have now explicitly reported that the overall response rate of the 2021 Iran STEPS survey was 97.73%.
2. In the Statistical Analysis section (Page 9, lines 199-200), we have added a sentence explicitly stating that design effect and cluster-based variance estimation were accounted for in all analyses.

2. They rely partly on self-reported diagnoses for CVD and cancer but laboratory or clinical cutoffs for diabetes, HTN, and CKD. This inconsistency could lead to differential misclassification bias across conditions. The authors should discuss this in the Discussion.

**Response:** We thank the reviewer for highlighting this important point regarding potential misclassification. To address this, we have added a sentence in the Discussion to clearly acknowledge this limitation. The added sentence reads: "It should also be noted that CVD and cancer were based on self-reported diagnoses, whereas HTN, CKD, and diabetes were identified through clinical or laboratory criteria. This difference in disease ascertainment may potentially lead to an underestimation of CVD and cancer and influencing the relative distribution of comorbidity types." (Page 18, Lines 315-319). This addition clarifies the potential impact of differing disease ascertainment methods on the reported comorbidity patterns and ensures transparency regarding this methodological limitation.

3. The authors note “complete-case analysis”. However, they should also describe the proportion or pattern of missingness for each variables, whether data were missing at random or not, or if any sensitivity analyses were performed. A table summarizing missing data by variable in appendix, and justification for using complete-case analysis are recommended.

**Response:** Thanks for the reviewer’s comment. Given that the missing data for covariates was <10% (maximum 5.1%) as shown in Fig S1, complete case analysis was employed, as the imputation may offer little advantage [1, 2] (Page 7, Lines 137-139). However, as a sensitivity analysis multiple imputation with 10 iteration was done and results remained unchanged (data not shown).

4. Why a multinomial rather than an ordered logistic model was chosen (the number of comorbidities is ordinal: 0, 1, ≥2)?

**Response:** We appreciate the reviewer's valuable question regarding the choice of our regression model. The choice of using multinomial stepwise logistic regression over ordinal logistic regression depends to the nature of the dependent variable. Ordinal logistic regression is appropriate when the dependent variable is ordinal by nature (e.g., categories that have a natural order). If the categories (0, 1, more than 2) were treated as nominal (without a natural order), multinomial logistic regression would be more suitable. In our cases there is no order between prevalence of HTN, CVD, CVD, or cancer. Moreover, Ordinal logistic regression assumes that the relationship between each pair of outcome groups is the same (the proportional odds assumption). While it’s not true in our data, therefore multinomial logistic regression may be preferred [3, 4].

Additionally, it is important to discuss if there is multicollinearity among predictors (e.g., BMI, waist circumference, physical activity, education)? If so, would it affect the author’s findings and conclusions?

**Response:** We have assessed multicollinearity among predictors in our models. All variance inflation factors (VIFs) were below 2, indicating no significant multicollinearity. You can find each variable’s VIF in the table below:

| Variable | Categories | VIF |
| --- | --- | --- |
| Age |  | 1.36 |
| Sex |  | 1.78 |
| Residential area |  | 1.19 |
| Years of schooling |  |  |
|  | 1_6 vs. illiterate | 1.71 |
|  | 7_11 vs. illiterate | 1.63 |
|  | ≥12 vs. illiterate | 1.98 |
| BMI |  |  |
|  | 25-30 vs. <25 | 1.90 |
|  | >30 vs. <25 | 1.97 |
| Marital status |  | 1.18 |
| Employment status |  | 1.58 |
| Physical activity |  | 1.06 |
| Wealth index |  |  |
|  | 2nd quintile vs. 1st quintile | 1.61 |
|  | 3rd quintile vs. 1st quintile | 1.63 |
|  | 4th quintile vs. 1st quintile | 1.79 |
|  | 5th quintile vs. 1st quintile | 1.96 |
| Smoking |  | 1.10 |
| Ever alcohol consumption |  | 1.05 |
| Basic health insurance |  | 1.02 |
| Glucometer at home |  | 1.09 |
| Diet score |  |  |
|  | 2nd tertile vs. 1st tertile | 1.39 |
|  | 3rd tertile vs. 1st tertile | 1.54 |

Given the logical and empirically observed collinearity between BMI and waist circumference, only BMI was included in the final model. Additionally, we have added a sentence in the Methods section to explicitly state that multicollinearity was checked using VIFs (Page 10, Lines 216-218).

5. The authors seem omit several plausible covariates available in Iran STEPS data. For example, they mention a “diet score” descriptively but not in the model. Did the data and the models include medication use beyond antihypertensives or glucose-lowering drugs?

**Response:** We appreciate the reviewer’s insightful comment. In this study, all variables available in the national STEPS survey that could plausibly be associated with our outcome among individuals with diabetes were considered. The diet score was also included as one of the candidate variables; as shown in Table 1, its distribution was examined descriptively. This variable was initially entered into the multinomial logistic regression model; however, it did not remain in the final multivariable model because it was not statistically significant in the stepwise selection procedure. Regarding medication use, only glucose-lowering medications and antihypertensive drugs were included, because other medications captured in STEPS were not relevant to the diseases under investigation in this study.

**References:**

[1]	P. Cummings, "Missing data and multiple imputation," (in eng), JAMA pediatrics, vol. 167, no. 7, pp. 656-61, Jul 2013, doi: 10.1001/jamapediatrics.2013.1329.

[2]	F. Barzi and M. Woodward, "Imputations of missing values in practice: results from imputations of serum cholesterol in 28 cohort studies," (in eng), American journal of epidemiology, vol. 160, no. 1, pp. 34-45, Jul 1 2004, doi: 10.1093/aje/kwh175.

[3]	C. Kwak and A. Clayton-Matthews, "Multinomial logistic regression," Nursing research, vol. 51, no. 6, pp. 404-410, 2002.

[4]	P. Warner, "Ordinal logistic regression," Journal of Family Planning and Reproductive Health Care, vol. 34, no. 3, p. 169, 2008.
