# Supplementary material for: Prevalence and factors associated with comorbidities in Iranian patients with type 2 diabetes: A national study
Source: PLoS One. 2026 Feb 27;21(2):e0343690. doi: 10.1371/journal.pone.0343690 (PMC12948068; doi:10.1371/journal.pone.0343690)
Supplement: Attachment — Submitted filename: Review Prevalence and Factors .docx [file pone.0343690.s007.docx]

**Review**

I reviewed the manuscript “**Prevalence and Factors Associated with Comorbidities in Iranian Patients with type 2 Diabetes: a national study**”. This manuscript addresses an important and timely issue—the prevalence and determinants of comorbidities among Iranian patients with Type 2 Diabetes Mellitus (T2DM). The study's focus on comorbidities such as hypertension, CKD, CVD, and cancer, and their association with sociodemographic factors, offers comprehensive insights into the burden of multimorbidity in this population.

The manuscript is within the scope of the journal, but certain main shortcomings should be addressed before as below:

- The manuscript should be revised for language accuracy, as it contains several grammatical errors.
- Currently, the manuscript lacks detailed prevalence estimates for individual comorbidities and their clustering patterns, which are crucial for comprehending the overall burden.
- Although the multinomial logistic regression approach is suitable, investigating interaction effects (e.g., between age and BMI or socioeconomic status and physical activity) could yield additional valuable insights.
- Conducting sensitivity analyses, such as stratification by age groups or adjusting for the duration of diabetes, might improve the depth of understanding.
- Consistent reporting of percentages and confidence intervals, along with correction of minor typographical errors, would enhance the manuscript's professionalism.
- The discussion should consider potential unmeasured confounders, such as medication adherence, healthcare access, or genetic factors, which could influence patterns of comorbidity and glycemic control.
- It could also expand on how these findings should shape healthcare delivery, emphasizing integrated care models, routine screening in primary care settings, and health education initiatives tailored to high-risk populations.
