# Supplementary material for: Prevalence and factors associated with comorbidities in Iranian patients with type 2 diabetes: A national study
Source: PLoS One. 2026 Feb 27;21(2):e0343690. doi: 10.1371/journal.pone.0343690 (PMC12948068; doi:10.1371/journal.pone.0343690)
Supplement: Attachment — Submitted filename: Response_to_Reviewers_auresp_3.docx [file pone.0343690.s009.docx]

**We would like to sincerely thank the reviewers for their careful reading of our manuscript and for their insightful and constructive comments. We have revised the manuscript accordingly, and our point-by-point responses are provided below.**

**Kind Regards**

**Dr. Nazila Rezaei,
Corresponding author**

**Title:** Prevalence and Factors Associated with Comorbidities in Iranian Patients with Diabetes: a national study

**Submission ID:** PONE-D-25-25729R1

**Review**

I reviewed the manuscript “**Prevalence and Factors Associated with Comorbidities in Iranian Patients with type 2 Diabetes: a national study**”. This manuscript addresses an important and timely issue—the prevalence and determinants of comorbidities among Iranian patients with Type 2 Diabetes Mellitus (T2DM). The study's focus on comorbidities such as hypertension, CKD, CVD, and cancer, and their association with sociodemographic factors, offers comprehensive insights into the burden of multimorbidity in this population.

The manuscript is within the scope of the journal, but certain main shortcomings should be addressed before as below:

- The manuscript should be revised for language accuracy, as it contains several grammatical errors.

**Response:** We thank the reviewer for highlighting the need to improve language accuracy in the manuscript. In response, we have thoroughly reviewed the entire article from the beginning and corrected all grammatical errors that were identified.

- Currently, the manuscript lacks detailed prevalence estimates for individual comorbidities and their clustering patterns, which are crucial for comprehending the overall burden.

**Response:** Thank you for the valuable comment. We have added the overall prevalence of each comorbidity in the entire cohort (HTN: 63.46%, CKD: 27.44%, CVD: 20.61%, Cancer: 1.88%). This, together with the clustering patterns already presented, now provides a complete picture of the comorbidity burden. The results section has been updated accordingly **(Page 11, lines 232-236).**

- Although the multinomial logistic regression approach is suitable, investigating interaction effects (e.g., between age and BMI or socioeconomic status and physical activity) could yield additional valuable insights.

**Response:** Thank you for your insightful comment and constructive feedback. As requested, we have examined the potential interactions between age and sex with the other covariates in our model. Since there were no significant interaction between sex, age and other covariates, all analysis was done in the whole population **(Page 10-11, lines 222-226)**.

- Conducting sensitivity analyses, such as stratification by age groups or adjusting for the duration of diabetes, might improve the depth of understanding.

**Response:** We thank the reviewer for the suggestion. In our primary analysis, we examined interactions between age, sex and other variables, none of which were statistically significant. Due to limited statistical power, a stratified analysis by age was not performed, and the results are presented in aggregate. Furthermore, to enhance the study results, we have included sex-stratified results in the supplementary material **(Table S1 & Table S2)**. Information on the duration of diabetes was not available in the dataset, which is noted in the Limitations section **(Page 23, line 420)**.

- Consistent reporting of percentages and confidence intervals, along with correction of minor typographical errors, would enhance the manuscript's professionalism.

**Response:** We thank the reviewer for pointing out the need for consistent reporting. We have ensured uniform formatting of percentages and confidence intervals throughout the manuscript and corrected all minor typographical errors.

- The discussion should consider potential unmeasured confounders, such as medication adherence, healthcare access, or genetic factors, which could influence patterns of comorbidity and glycemic control.

**Response:** Thank you for this insightful comment. We have revised the Discussion section to explicitly acknowledge the potential impact of unmeasured confounders, including medication adherence, access to healthcare services, treatment intensity, and genetic predisposition, on comorbidity patterns and glycaemic control. We also noted that the lack of these variables may result in residual confounding and should be considered when interpreting the findings **(Page 21, lines 345-350)**.

- It could also expand on how these findings should shape healthcare delivery, emphasizing integrated care models, routine screening in primary care settings, and health education initiatives tailored to high-risk populations.

**Response:** Thank you for this valuable suggestion. We have revised the Conclusion to explicitly highlight the implications of our findings for healthcare delivery, emphasizing the role of integrated care models within primary care settings and routine screening for comorbidities among high-risk populations **(Page 26, lines 458-460).**
